# Supplementary material for: Characteristics of wild hazelnut populations in Northeast China and selection of superior provenances
Source: PLoS One. 2024 Dec 3;19(12):e0313954. doi: 10.1371/journal.pone.0313954 (PMC11614255; doi:10.1371/journal.pone.0313954)
Supplement: S1 File — (PDF) [file pone.0313954.s005.pdf]

青岛斯坦德标准检测有限公司

项 目 报 告

报告编号	GN-20230911-007N
委托单位	齐齐哈尔大学
委托地址	齐齐哈尔市龙沙区翡翠华庭小区 6 栋二单元 3102 室

DRAFT

2023 年 09 月 18 日

测试服务条款

1、　本报告无本公司检测专用章、骑缝章无效。

2、　本报告部分复制、私自转让、盗用、冒用、涂改、增删或以其他形式篡改，均属无效，且本公司将追究上述行为的法律责任。

3、　本报告仅用于科研、教学、内部质量控制等活动，不具有社会证明作用，未经本公司允许不得将此报告用于法庭举证、仲裁及其他相关活动。

4、　未经本公司允许，不得将本报告及本公司名称用于产品标签、广告、评优及商品宣传等活动。

5、　除非另有说明，本报告仅对所检样品负责。

6、　如样品为委托方送检时，委托单位对样品的代表性和所提供的样品信息、资料的真实性负责，本公司不承担任何相关责任。

7、　对本报告若有异议，应于收到报告之日起五日内向本公司提出。

DRAFT

检验地点：山东省青岛市城阳区丰茂路 55 号斯坦德集团总部

电　　话：0532-58938059

邮政编码：266000

1.样品信息

样品信息 ： 榛仁 A、榛仁 CK，规格：带壳干果，批号：2023 年 9 月

样品数量 ： 30 克/样

收样日期 ： 2023/09/11

测试周期 ： 2023/09/11~2023/09/15

测试要求 ： 按照客户要求测试

2.检测指标

本次检测项目：可溶性糖、粗脂肪、粗蛋白、维生素 C、17 种水解氨基酸、K、Ca、Mg、Fe、Zn

3.检测结果

项目	单位	1	2
		**榛仁 A**	**榛仁 CK**
		**来自野生优良单株的多个子代结实样品**	**来自当地乡土树种多个植株的果实混合样品**
天冬氨酸（Asp）	g/100g	1.68	1.71
苏氨酸（Thr）	g/100g	0.51	0.52
丝氨酸（Ser）	g/100g	0.69	0.69
谷氨酸（Glu）	g/100g	3.48	3.57
甘氨酸（Gly）	g/100g	0.77	0.77
丙氨酸（Ala）	g/100g	0.82	0.80
胱氨酸（Cys）	g/100g	0.21	0.23
缬氨酸（Val）	g/100g	0.85	0.83
蛋氨酸（Met）	g/100g	0.13	0.15
异亮氨酸（IIe）	g/100g	0.68	0.67
亮氨酸（Leu）	g/100g	1.16	1.15
酪氨酸（Tyr）	g/100g	0.46	0.46

DRAFT

项目	单位	1	2
		**榛仁 A**	**榛仁 CK**
		**来自野生优良单株的多个子代结实样品**	**来自当地乡土树种多个植株的果实混合样品**
苯丙氨酸（Phe）	g/100g	0.75	0.77
赖氨酸（Lys）	g/100g	0.53	0.56
组氨酸（His）	g/100g	0.44	0.42
精氨酸（Arg）	g/100g	2.95	2.82
脯氨酸（Pro）	g/100g	0.53	0.53
可溶性糖	%	7.07	7.18
粗脂肪	g/100g	51.6	45.9
粗蛋白	g/100g	24.7	25.6
维生素 C	mg/kg	1.53	1.23
K	mg/kg	6.24×10^{3}	7.89×10^{3}
Ca	mg/kg	2.07×10^{3}	2.49×10^{3}
Mg	mg/kg	2.46×10^{3}	2.67×10^{3}
Fe	mg/kg	48.3	57.4
Zn	mg/kg	55.5	56.3
备注	--		

本表格结束

以上系申请人自送样品的测试结果，其结果仅对来样负责

DRAFT

4.检测方法（一）

水解氨基酸测试方法-氨基酸自动分析仪

4.1 设备基本信息

设备名称	厂家	型号
电子天平	上海舜宇恒平科学仪器有限公司	FA-1004
离心机	湘仪	TGL-16M
氨基酸自动分析仪	日本株式会社日立高新技术科学	LA8080

4.2 试剂、标品信息

试剂/标品	厂家
氨基酸混标	和光纯药工业株式会社
盐酸	国药集团化学试剂有限公司
柠檬酸钠	国药集团化学试剂有限公司
氢氧化钠	国药集团化学试剂有限公司

4.3 测试方法

4.3.1 前处理方法

称取混合均匀的适量样品，在水解管中加入 10mL 1：1 盐酸溶液，混匀，将水解管放在 110℃±1℃的电热鼓风恒温箱中水解 22h 后，取出，冷却至室温。打开水解管，将水解液过滤至 25mL 容量瓶中，用少量水多次冲洗水解管，水洗液移入同一 25mL 容量瓶内，最后用水定容至刻度，摇匀。准确吸取 0.5mL 滤液移入至 15mL 试管内，4 氮气吹干，用 0.02mol/L 盐酸溶液定容至 10ml，振荡混匀后，过 0.22μm 微孔滤膜后，上机测定。

4.3.2 仪器方法

（1）色谱柱：磺酸型阳离子树脂；

（2）波长：570nm 和 440nm；

（3）进样量：20μL，流速：见具体方法；

（4）反应温度：135±5℃；

（5）流动相比例：

DRAFT

Time（min）	%B1	%B2	%B3	%B4	%B5	%B6	Flow（mL/min）	temp	%R1	%R2	%R3	Flow（mL/min）
0.0	100	0	0	0	0	0	0.400	57	50	50	0	0.350
2.5	100	0	0	0	0	0						
2.6	0	100	0	0	0	0						
4.5	0	100	0	0	0	0						
4.6	0	0	100	0	0	0						
12.8	0	0	100	0	0	0						
12.9	0	0	0	100	0	0						
27.0	0	0	0	100	0	0						
27.1	0	0	0	0	0	100						
32.0									50	50	0	
32.1									0	0	100	
33.0	0	0	0	0	0	100						
33.1	0	100	0	0	0	0						
34.0	0	100	0	0	0	0						
34.1	100	0	0	0	0	0						
37.0									0	0	100	
37.1									50	50	0	
53.0	100	0	0	0	0	0						

4.4 计算公式

样本中各氨基酸含量：$X=\frac{(C-C_0)*V*N}{m*10000}$

公式中各字母含义：

X——样品中各氨基酸的含量，单位为g/100g；

C——样品中各氨基酸根据标准品浓度计算所得的浓度值，单位为mg/L；

C_0——空白对照中各氨基酸根据标准品浓度计算所得的浓度值，单位为mg/L；

V——定容体积，单位为mL；

N——稀释倍数；

m——称样量，单位为g。

5.参考文献

[1]GB 5009.124-2016 食品安全国家标准 食品中氨基酸的测定

[2]Lin H, Yu X, Fang J, et al. Flavor Compounds in Pixian Broad-Bean Paste: Non-Volatile Organic Acids and Amino Acids. Molecules. 2018;23(6):1299. Published 2018 May 29.

DRAFT

4.检测方法（二）

粗脂肪测试方法

4.1 设备基本信息

设备名称	厂家	型号
电子天平	上海舜宇恒平科学仪器有限公司	舜宇恒平 FA-1004
恒温水浴锅	上海双捷实验设备有限公司	DRHH-S6
粗脂肪测定仪	上海新嘉电子有限公司	SZF-06A

4.2 试剂、标品信息

试剂名称	厂家
无水乙醚	国药集团化学试剂有限公司
石油醚	天津市富宇精细化工有限公司

4.3 测试方法

称取混匀后的适量样品，全部移入滤纸筒内。将滤纸筒放入索氏抽提器的抽提筒内，连接已干燥至恒重的接收瓶，由抽提器冷凝管上端加入无水乙醚或石油醚至瓶内容积的三分之二处，于水浴上加热，使无水乙醚或石油醚不断回流抽提(6 次/h8 次/h)，一般抽提 6h~10h。提取结束时，用磨砂玻璃棒接取 1 滴提取液，磨砂玻璃棒上无油斑表明提取完毕。取下接收瓶，回收无水乙醚或石油醚，待接收瓶内溶剂剩余 1mL~2mL.时在水浴上蒸干，再于 100℃±5℃干燥 1h，放干燥器内冷却 0.5h 后称量。重复以上操作直至恒重(直至两次称量的差不超过 2mg)。

4.4 计算公式

食品中脂肪含量按公式计算： $X = \frac{m_1 - m_0}{m_2} \times 100$

式中：

X——试样中脂肪的含量，单位为克每百克（g/100g）。

m_1——恒重后接受瓶和脂肪的含量，单位为克（g）。

m_0——接收瓶的质量，单位为克（g）。

m_2——试样的质量，单位为克（g）

5.参考文献

[1]参考GB 5009.6-2016 食品安全国家标准 食品中脂肪的测定

DRAFT

4.检测方法（三）

可溶性糖测试方法

4.1 设备基本信息

设备名称	厂家	型号
电子天平	上海舜宇恒平科学仪器有限公司	FA-1004
紫外分光光度计	Agilent	8453

4.2 试剂、标品信息

试剂名称	厂家
葡萄糖	上海源叶生物科技有限公司
DNS	国药集团

4.3 测试方法

称取 0.5g 左右打碎混匀试样，加入 40mL 超纯水，亚铁氰化钾和乙酸锌各 1mL，摇匀，加超纯水定容至 50mL，放置片刻，过滤。取滤液 1mL，加入 6mol/L 盐酸 1mL，80℃加热 10min，取出，冷水冷却至室温，加甲基红指示剂 3 滴，用 6mol/L 氢氧化钠溶液中和至浅橙色，用水定容至 50mL，摇匀。取滤液 2mL 加入 DNS 试剂 4mL，沸水浴 5min，取出，冷却到室温，用水定容至 10mL，摇匀。于 540nm 处测吸光值。

标曲建立：移液枪精确吸取 0mL、0.2mL、0.4mL、0.6mL、0.8mL、1.0mL 的标准葡萄糖工作溶液置于 6 支 10mL 具塞试管中，用蒸馏水补至 2.0mL。加入 DNS 试剂 4mL，置于沸水浴 5min。取出冷却至室温，定容，摇匀。分光光度计 540nm 测吸光度。以葡萄糖浓度为横坐标，吸光度值为纵坐标，制定标准曲线。

4.4 计算公式

可溶性糖含量按公式计算：

$$X=\frac{m_1*v_1*0.001*100}{m*v_2}$$

X——试样中可溶性糖的含量，单位为%；

V_1——处理液总体积，单位为 mL；

V_2——测定用样液体积，单位为 mL；

m——称样量，单位为 g；

m_1——可溶性糖（以葡萄糖计）的质量，单位为 mg。

5.参考文献

[1]参考 NY/T 1278-2007 蔬菜及其制品中可溶性糖的测定 铜还原碘量法

[2]参考 NY/T 2742-2015 水果及制品可溶性糖的测定 3,5-二硝基水杨酸比色法

DRAFT

4.检测方法（四）

蛋白质的测试方法

4.1 设备基本信息

设备名称	厂家	型号
电子天平	上海舜宇恒平科学仪器有限公司	FA-1004
凯氏定氮仪	济南海能仪器股份有限公司	K9840

4.2 试剂、标品信息

试剂/标品	厂家
硫酸铜	国药集团化学试剂有限公司
硫酸钾	国药集团化学试剂有限公司
硫酸	国药集团化学试剂有限公司
硼酸	国药集团化学试剂有限公司
甲基红指示剂	国药集团化学试剂有限公司
溴甲酚绿指示剂	国药集团化学试剂有限公司
盐酸标准滴定溶液	国药集团化学试剂有限公司

4.3 测试方法

4.3.1 甲基红乙醇溶液（1g/L）

称取 0.1g 甲基红，溶于 95%乙醇，用 95%乙醇稀释至 100mL。

4.3.2 溴甲酚绿乙醇溶液（1g/L）

取 0.1g 溴甲酚绿，溶于 95%乙醇，用 95%乙醇稀释至 100mL。

称取适量样品于消化管中，加入 0.4g 硫酸铜、6g 硫酸钾及 12mL 硫酸于消化炉进行消化，当消化炉温度达到 420℃之后，此时消化管中的液体呈蓝绿色等澄清透明后，继续消化 1h，取出冷却后加入 20mL 水，放冷，于自动凯氏定氮仪上蒸馏 7min，接收瓶中加指示剂混合溶液 1-2 滴(1 份甲基红乙醇溶液与 5 份溴甲酚绿乙醇溶液现配现用）和 10mL 硼酸溶液（20g/L），接收蒸馏液到 200mL，然后用盐酸标准溶液（0.100mol/L）滴定，终点为浅灰红色，同时做试剂空白。

4.4 计算公式

DRAFT

蛋白质含量按公式计算：$X=\frac{(V_1-V_2)\times c\times 0.0140}{m\times V_3/100}\times F\times 100$

式中：

X——试样中蛋白质的含量，单位为 g/100g；

V_1——试样消耗盐酸标准滴定溶液的体积，单位为 mL；

V_2——试剂空白消耗盐酸标准滴定溶液的体积，单位为 mL；

V_3——吸取消化液的体积，单位为 mL；

c——盐酸标准滴定溶液的浓度，单位为 mol/L；

m——称样量，单位为 g；

F——氮换算成蛋白质的系数，具体参见下表。

表 A.1 蛋白质折算系数表

食品类别		折算系数	食品类别		折算系数
小麦	全小麦粉	5.83	大米及米粉		5.95
	麦糠麸皮	6.31	鸡蛋	鸡蛋(全)	6.25
	麦胚芽	5.80		蛋黄	6.12
	麦胚粉、黑麦、普通小麦、面粉	5.70		蛋白	6.32
燕麦、大麦、黑麦粉		5.83	肉与肉制品		6.25
小米、裸麦		5.83	动物明胶		5.55
玉米、黑小麦、饲料小麦、高粱		6.25	纯乳与纯乳制品		6.38
油料	芝麻、棉籽、葵花籽、蓖麻、红花籽	5.30	复合配方食品		6.25
	其他油料	6.25	酪蛋白		6.40
	菜籽	5.53			
坚果、种子类	巴西果	5.46	胶原蛋白		5.79
	花生	5.46	豆类	大豆及其粗加工制品	5.71
	杏仁	5.18		大豆蛋白制品	6.25
	核桃、榛子、椰果等	5.30	其他食品		6.25

5.参考文献

[1]参考 GB 5009.5-2016 食品安全国家标准 食品中蛋白质的测定

[2]参考 GB/T 6432-2018 饲料中粗蛋白的测定 凯氏定氮法

4.检测方法（五）

元素测试方法-MS

4.1 设备基本信息：

设备名称	厂家	型号
电子天平	上海舜宇恒平科学仪器有限公司	舜宇恒平 FA-1004
微波消解仪	上海屹尧仪器科技发展有限公司	TOPEX
电感耦合等离子体质谱仪	美国 thermo 公司	iCAPRQ

4.2 试剂、标品信息：

试剂/标品	厂家
硝酸	国药集团化学试剂有限公司
元素标准溶液	国家有色金属及电子材料分析测试中心

4.3 测试方法：

称取适量样品至聚四氟乙烯消解罐中，加入 5 mL 硝酸。静置，反应结束后，盖盖密封，放入微波消解仪，消解程序见下表。

步骤	温度（℃）	保温时间（min）
1	100	3
2	140	3
3	160	3
4	180	3
5	190	15

待温度冷却至 50℃以下后，取出消解罐放入通风橱中，打开消解罐，用超纯水润洗，转移至 25mL 容量瓶中，至少润洗 3~4 次，用超纯水稀释定容至刻度，待测。空白对照同法处理。

ICP-MS 仪器参数如下：

ICP-MS Parameter	Value
RF power（射频功率）	1550 W
Pump Speed（泵速）<br>S/C temperature（雾化室温度）	40rpm<br>2.7 ℃

DRAFT

ICP-MS Parameter	Value
Smpl Depth（采样深度）	5 mm
Cool flow（冷却气流速）	14 L/min
Auxilliary flow（辅助气流速）	0.8 L/min
Nebulizer flow(雾化气流速）	1.122L/min

4.4 计算公式：

元素含量按公式计算： $W=\frac{(C-C_0)*V*N}{m}$

式中：

W——试样中目标物含量，单位为 mg/kg；

C——试样测定液中目标物的浓度，单位 mg/L；

C_0——空白对照中目标物的浓度，单位 mg/L；

V——定容体积，单位 mL；

N——稀释倍数；

m——试样的取样量，单位为 g。

5.参考文献

[1]参考 GB 5009.268-2016 食品安全国家标准 食品中多元素的测定.

DRAFT

4.检测方法（六）

维生素 C 测试方法

4.1 设备基本信息

设备名称	厂家	型号
电子天平	上海舜宇恒平科学仪器有限公司	舜宇恒平 FA-1004
离心机	湘仪	TGL-16M
液相色谱仪	Thermo	U3000

4.2 试剂、标品信息

试剂名称	厂家
偏磷酸	国药集团化学试剂有限公司
甲醇	默克
十六烷基三甲基溴化铵	天津博迪化工股份有限公司
维生素 C	上海源叶生物科技有限公司

4.3 测试方法

4.3.1 前处理方法

称取混合均匀的适量样品于 50mL 容量瓶中，加入 40mL 20g/L 偏磷酸溶液，超声30min，定容至 50mL，过 0.22μm 微孔滤膜，上机检测。

4.3.2 仪器方法

（1）色谱柱：SHISEIDO C_{18}（4.6mm*250mm*5μm）；

（2）检测器：DAD 检测器；

（3）柱温：25℃；

（4）进样量：10μL；

（5）流速：1.0mL/min；

（6）波长：245nm；

（7）VC 流动相：6.8L 的磷酸二氢钾和 0.91g 十六烷基三甲基溴化铵，用水溶解并定容至 1L（用磷酸调 pH2.5-2.8），超声脱气；

（8）流动相 A：甲醇；流动相 B：VC 流动相；

DRAFT

（9）流动相比例：A：B=2：98。

4.4 计算公式

维生素 C 含量按公式计算： $W=\frac{(C-C_0)*V*N}{m}$

式中：

W——试样中目标物含量，单位 mg/kg；

C——试样测定液中目标物的浓度，单位 mg/L；

C_0——空白对照中目标物的浓度，单位 mg/L；

V——定容体积，单位 mL；

N——稀释倍数；

m——试样的取样量，单位为 g。

5.参考文献

[1]参考 Study on the determination of L-ascor bic acid in orange by high performance liquid chromatography[J]. Food Research and Development,2018,39(4):148-151.

[2]参考 RP-HPLC determination of nine water-soluble vitamins[J]. Chinese Journal of Pharmaceutical Analysis,2005,25(3):339-341.

[3]参考 GB 5009.86-2016 食品安全国家标准食品中抗坏血酸的测定

---报告结束---

DRAFT
