# Supplementary material for: Characteristics of wild hazelnut populations in Northeast China and selection of superior provenances
Source: PLoS One. 2024 Dec 3;19(12):e0313954. doi: 10.1371/journal.pone.0313954 (PMC11614255; doi:10.1371/journal.pone.0313954)
Supplement: Attachment — Submitted filename: Response to Reviewers.docx [file pone.0313954.s006.docx]

**Dear Editors and Reviewers,**

Thank you for your letter and for the reviewer’s comments concerning our manuscript entitled “Characteristics of wild hazelnut populations in northeast China and selection of superior provenances” (ID: PONE-D-24-23349).Those comments are all valuable and very helpful for revising and improving our paper, as well as the important guiding significance to our researches. We have studied comments carefully and have made correction which we hope meet with approval. Furthermore, we would like to show the details as follows:

**Academic Editor：**

*1. When submitting your revision, we need you to address these additional requirements.*

*Please ensure that your manuscript meets PLOS ONE's style requirements, including those for file naming. The PLOS ONE style templates can be found at https://journals.plos.org/plosone/s/file?id=wjVg/PLOSOne_formatting_sample_main_body.pdf and* [*https://journals.plos.org/plosone/s/file?id=ba62/PLOSOne_formatting_sample_title_authors_affiliations.pdf*](https://journals.plos.org/plosone/s/file?id=ba62/PLOSOne_formatting_sample_title_authors_affiliations.pdf)

**The author’s answer:**

We have read the style templates carefully and have revised the manuscript to PLOS ONE's style, which we hope will meet your requirements. If there are any questions, please point them out and we will make corrections soon.

*2. Thank you for stating the following financial disclosure:*

*This research was funded by Basic Research Fees of Universities in Heilongjiang Province, China (145309628) (https://kygl.webvpn.qqhru.edu.cn/userAction!do_casLogin.action).*

*Please state what role the funders took in the study. If the funders had no role, please state: "The funders had no role in study design, data collection and analysis, decision to publish, or preparation of the manuscript."*

*If this statement is not correct you must amend it as needed.*

*Please include this amended Role of Funder statement in your cover letter; we will change the online submission form on your behalf.*

**The author’s answer:**

Thank you for bringing that up. This research was funded by the Basic Research Fees of Universities in Heilongjiang Province, China (145309628), Graduate Innovation Project of Qiqihar University (X202310232032), Graduate Innovation Project of Qiqihar University (X202310232040). What role the funders took in the study: All funders provided financial support.

*3. We note that your Data Availability Statement is currently as follows: All relevant data are within the manuscript and its Supporting Information files.*

*Please confirm at this time whether or not your submission contains all raw data required to replicate the results of your study. Authors must share the “minimal data set” for their submission. PLOS defines the minimal data set to consist of the data required to replicate all study findings reported in the article, as well as related metadata and methods (https://journals.plos.org/plosone/s/data-availability#loc-minimal-data-set-definition).*

*For example, authors should submit the following data:*

*- The values behind the means, standard deviations and other measures reported;*

*- The values used to build graphs;*

*- The points extracted from images for analysis.*

*Authors do not need to submit their entire data set if only a portion of the data was used in the reported study.*

*If your submission does not contain these data, please either upload them as Supporting Information files or deposit them to a stable, public repository and provide us with the relevant URLs, DOIs, or accession numbers. For a list of recommended repositories, please see https://journals.plos.org/plosone/s/recommended-repositories.*

*If there are ethical or legal restrictions on sharing a de-identified data set, please explain them in detail (e.g., data contain potentially sensitive information, data are owned by a third-party organization, etc.) and who has imposed them (e.g., an ethics committee). Please also provide contact information for a data access committee, ethics committee, or other institutional body to which data requests may be sent. If data are owned by a third party, please indicate how others may request data access.*

**The author’s answer:**

Thank you for bringing that up. We submit the minimal data set on the supporting information that contains the values behind the means, standard deviations, the values used to build graphs and the points extracted from images for analysis.

*4. When completing the data availability statement of the submission form, you indicated that you will make your data available on acceptance. We strongly recommend all authors decide on a data sharing plan before acceptance, as the process can be lengthy and hold up publication timelines. Please note that, though access restrictions are acceptable now, your entire data will need to be made freely accessible if your manuscript is accepted for publication. This policy applies to all data except where public deposition would breach compliance with the protocol approved by your research ethics board. If you are unable to adhere to our open data policy, please kindly revise your statement to explain your reasoning and we will seek the editor's input on an exemption. Please be assured that, once you have provided your new statement, the assessment of your exemption will not hold up the peer review process.*

**The author’s answer:**

Thank you for your advice. We decide on a data sharing plan before acceptance.

*5. Please amend either the title on the online submission form (via Edit Submission) or the title in the manuscript so that they are identical.*

**The author’s answer:**

Thank you for pointing this out. We have amended the title on the online submission.

*6. We note that Figure 1 in your submission contain map images which may be copyrighted. All PLOS content is published under the Creative Commons Attribution License (CC BY 4.0), which means that the manuscript, images, and Supporting Information files will be freely available online, and any third party is permitted to access, download, copy, distribute, and use these materials in any way, even commercially, with proper attribution. For these reasons, we cannot publish previously copyrighted maps or satellite images created using proprietary data, such as Google software (Google Maps, Street View, and Earth). For more information, see our copyright guidelines: http://journals.plos.org/plosone/s/licenses-and-copyright.*

*We require you to either present written permission from the copyright holder to publish these figures specifically under the CC BY 4.0 license, or remove the figures from your submission:*

*a. You may seek permission from the original copyright holder of Figure 1 to publish the content specifically under the CC BY 4.0 license.*

*We recommend that you contact the original copyright holder with the Content Permission Form (http://journals.plos.org/plosone/s/file?id=7c09/content-permission-form.pdf) and the following text:*

*“I request permission for the open-access journal PLOS ONE to publish XXX under the Creative Commons Attribution License (CCAL) CC BY 4.0 (http://creativecommons.org/licenses/by/4.0/). Please be aware that this license allows unrestricted use and distribution, even commercially, by third parties. Please reply and provide explicit written permission to publish XXX under a CC BY license and complete the attached form.”*

*Please upload the completed Content Permission Form or other proof of granted permissions as an "Other" file with your submission.*

*In the figure caption of the copyrighted figure, please include the following text: “Reprinted from [ref] under a CC BY license, with permission from [name of publisher], original copyright [original copyright year].”*

*b. If you are unable to obtain permission from the original copyright holder to publish these figures under the CC BY 4.0 license or if the copyright holder’s requirements are incompatible with the CC BY 4.0 license, please either i) remove the figure or ii) supply a replacement figure that complies with the CC BY 4.0 license. Please check copyright information on all replacement figures and update the figure caption with source information. If applicable, please specify in the figure caption text when a figure is similar but not identical to the original image and is therefore for illustrative purposes only.*

*The following resources for replacing copyrighted map figures may be helpful:*

*USGS National Map Viewer (public domain): http://viewer.nationalmap.gov/viewer/*

*The Gateway to Astronaut Photography of Earth (public domain): http://eol.jsc.nasa.gov/sseop/clickmap/*

*Maps at the CIA (public domain): https://www.cia.gov/library/publications/the-world-factbook/index.html and https://www.cia.gov/library/publications/cia-maps-publications/index.html*

*NASA Earth Observatory (public domain): http://earthobservatory.nasa.gov/*

*Landsat: http://landsat.visibleearth.nasa.gov/*

*USGS EROS (Earth Resources Observatory and Science (EROS) Center) (public domain): http://eros.usgs.gov/#*

*Natural Earth (public domain): http://www.naturalearthdata.com/*

**The author’s answer:**

We have tried our best to obtain permission from the original copyright holders, but to no avail. We have therefore decided to remove the figure. We have also changed the numbering of the figures in the manuscript.

*7. Please remove your figures from within your manuscript file, leaving only the individual TIFF/EPS image files, uploaded separately. These will be automatically included in the reviewers’ PDF.*

**The author’s answer:**

Thank you for bringing that up. We have removed all figures from within manuscript file.

**Reviewer #1:**

**Shortcomings of the abstract of the paper：**

***1. Lack of clarity on the research methodology:*** *The abstract do not provide detailed information on the specific methodology used in the provenance trials and genetic analysis of the hazelnut species. Without a clear explanation of the experimental design, data collection methods, and statistical analysis, it is difficult for readers to assess the reliability and validity of the study's findings.*

**The author’s answer:**

We thank the reviewer for pointing this out. We have provided detailed information on the specific methodology used in the provenance trials and genetic analysis of the hazelnut species. We have changed [This study addresses this knowledge gap by initiating provenance trials on eight distinctive wild hazelnut taxa, sourced from a broad array of seven different locations within Northeast China. The trials meticulously explored the trees' flowering biology, growth parameters, and the economic attributes of the produced nuts.] to [To develop and utilize high-quality wild hazelnut resources, this study selected eight populations of wild hazelnuts from seven different provenances in Heilongjiang Province, China. Natural hybrid seeds of eight populations were sown in the Chohai Forest Farm in Longjiang County, Heilongjiang Province, in 2018. In April 2020, two-year-old seedlings were used to establish a provenance trial forest, thereby initiating the provenance trial. Growth parameters were measured using electronic calipers, and pollen characteristics were observed under an electron microscope. The trials meticulously explored the trees' flowering biology, growth parameters, and the economic attributes of the produced nuts. Principal component analysis was employed to comprehensively assess differences among the wild hazelnut populations from various provenances, aiming to identify superior wild hazelnut provenances.] (page 1, line 4-13).

**Shortcomings of the Introduction of the paper：**

***1. Limited context and background information:*** *While the introduction briefly touches upon the ecological and economic significance of wild hazelnut species in China, it lacks a comprehensive review of existing literature on hazelnut or other nut trees breeding, genetics, and cultivation practices. Providing more context and background information would help situate the study within the broader research landscape and highlight the novelty and significance of the findings.*

**The author’s answer:**

Thank you for your suggestions! The references you suggested are benefit for us. We have read all references and learn a lot. In response to your suggestions, we have included content on the breeding, genetics, and cultivation practices of hazelnuts and other nut trees to provide a more comprehensive. Please see page 3 of the revised manuscript, line 47-63. We hope that it is now clearer.

***2. Absence of comparative analysis:*** *The introduction emphasizes the geographical variations and unique characteristics of the different provenances of wild hazelnut species in Northeast China. However, there is a lack of comparative analysis or discussion on how these variations may impact the hazelnuts' overall productivity, adaptability, and market value. A more thorough comparative analysis would enhance the depth of the study and provide stronger insights for potential breeding and cultivation strategies.*

**The author’s answer:**

Thank you for this valuable comments. We have read all references and learn a lot. As suggested by the reviewer, we have added content on how these variations may impact the hazelnuts' overall productivity, adaptability, and market value. Please see page 5 of the revised manuscript, line 93-101.

***3. Limited discussion on sustainability and future implications:*** *While the introduction briefly mentions the industrial and medicinal benefits of hazelnuts, there is a lack of discussion on the sustainability aspects of hazelnut cultivation and the potential long-term implications of the study's findings. Including a section on the environmental sustainability, socio-economic impact, and future research directions would enrich the paper and demonstrate a broader perspective on the importance of wild hazelnut biodiversity conservation and utilization.*

**The author’s answer:**

Thank you for highlighting this gap in our manuscript. We have added the suggested content to the manuscript on page 2 of the revised manuscript, line 42-46.

**Shortcomings of the Materials and Methods of the paper:**

***1. Lack of detail on sampling methodology:*** *The section briefly mentions the collection of hazelnut seeds from various provenances within Northeast China, but it does not provide specific details on the sampling strategy employed. Information on how the seeds were collected, the criteria used for selecting specific provenances, and the rationale behind the selection of the eight hazelnut species could strengthen the transparency and reproducibility of the study.*

**The author’s answer:**

We think this is an excellent suggestion. We provide specific details on the sampling strategy employed:

(1) How the seeds were collected: Thirty dominant trees were selected from each geographical source to serve as seed trees, and their natural hybrid seeds were collected (page 6, line 118-120).

(2) The criteria used for selecting specific provenances: For this experiment, seed trees were selected based on criteria that included the absence of severe pests and diseases and higher productivity (page 6, line 113-114).

(3) The rationale behind the selection of the eight hazelnut species：These species are only found in Heilongjiang Province and have some excellent traits. We have changed [Eight hazelnut species from seven different provenance sources within Northeast China were selected for this experiment] to [Eight hazelnut populations with some excellent traits distributed in seven different geographic provenance sources within the Heilongjiang Province were selected] (page 6, line 115-116).

***2. Insufficient information on experimental design:*** *While the section mentions the establishment of provenance trial forests with a randomized complete block design and three repeats, it lacks explicit details on the allocation of treatments, the randomization process, and the specific variables measured in the study. Providing a more comprehensive description of the experimental design, including the control measures implemented, could aid in better understanding the reliability and validity of the results obtained.*

**The author’s answer:**

Thank you for your valuable suggestions. We added a more comprehensive description of the experimental design. We have changed [Seedlings were nurtured in the Cuohai Forest Farm in Longjiang County to create provenance trial forests in April 2020 using 2-year old seedlings. The provenance trial forests were designed in a randomized complete block design with three repeats, and the number of rows of various provenance ranged from 1-12 rows in each repeat, with 10 plants in each row] to [Seedlings were nurtured in the Cuohai Forest Farm in Longjiang County, Heilongjiang province (E122˚24’55’’~122˚56’50’’, N47˚30’03’’ ~ 47˚14’55’’), to create provenance trial forests in April 2020 using 2-year old seedlings. The provenance trial forests was 0.67hm2 and a randomized complete block design was used to design four blocks with three replications, in each replication the number of rows of each provenance varied from 1-12 rows with 10 plants per row and a spacing of 1.5m x 3m] (page 6, line 120-125).

***3. Limited explanation of data analysis techniques:*** *The section briefly mentions the use of statistical analysis tools such as principal component analysis (PCA) and Microsoft Excel for processing and analyzing the data. However, it would be beneficial to include more information on the specific statistical tests used, how the data were managed and cleaned, and any assumptions underlying the analyses. This would provide readers with insights into the robustness of the statistical findings and interpretations.*

**The author’s answer:**

Thank you for these valuable suggestions. We have changed [The raw experimental data underwent processing and statistical analysis utilizing Microsoft Excel and IBM SPSS Statistics version 19.0. Graphical representations of the findings were generated using Microsoft Excel complemented by Origin 2024 software] to [The data were standardized prior to performing principal component analysis. The experimental data were analyzed using Microsoft Excel and IBM SPSS Statistics 19.0 to determine means, standard deviations, and significance levels. Graphs and charts were created using Microsoft Excel and Origin 2024] (page 10, line 182-185).

***4. Lack of discussion on potential biases and limitations:*** *The section does not address any potential biases or limitations that may have affected the study's outcomes. Acknowledging potential sources of bias, such as environmental variability, sampling errors, or measurement inconsistencies, could help contextualize the results and offer insights into the generalizability of the findings.*

**The author’s answer:**

Thank you very much for your suggestion. We have added potential biases or limitations of the study that may have affected the study's outcomes to the discussion：This study conducted a comprehensive evaluation to identify superior provenances, although certain limitations remain. The analysis of variance (ANOVA) employed in this experiment included both within-group and between-group variances to assess differences in various hazelnut characteristics—such as florescence, pollen sub-microstructure, growth parameters, and economic traits—among different provenance. Intergroup variation primarily resulted from genetic differences among geographic provenance sources, which were statistically represented as variation between groups. These genetic differences significantly impacted hazelnut performance, providing guidance for the selection of superior provenance sources and improved varieties. In contrast, intra-group variation was predominantly attributed to individual differences within the same geographic provenance, likely due to environmental factors. Within-group variation indicates that even within the same geographic provenance, individual differences affect hazelnut characteristics. This suggests that individual differences must be considered in variety selection and cultivation management (page 25, line 425-436).

**Reviewer #2:**

*1. Were they selected from the population or were the characteristics of these species already known? If there was a population, how was the selection of the population? What was the size of the population (how many hectares).*

**The author’s answer:**

Thank you for bringing up the issue. The seed trees selected for our provenance trials were chosen from the population based on their freedom from significant pests and diseases, as well as their high productivity. The size of the population was 0.67hm^2^.

*2. One-year measurement data is not reliable and should be repeated at least two or three years for morphological and phenological indicators.*

**The author’s answer:**

Thank you for your valuable feedback on the duration of data collection. We acknowledge that data reliability can be influenced by the duration of observations, and your suggestion to extend the measurement period is well-founded. Our study used one-year data mainly due to time constraints Despite this, we made every effort to ensure that our data collection and analysis were as robust as possible. We employed rigorous methodologies and controls to minimize errors and biases in our measurements. We also refer to [Zhang Q, Chen S, Qu G, Yang Y, Lu Z, Wang J, et al. (2024) Provenance and family variations in early growth of Manchurian walnut (Juglans mandshurica Maxim.) and selection of superior families. PLoS ONE 19(3): e0298918. https://doi.org/10.1371/journal.pone.0298918] that have demonstrated that one-year data can provide valuable insights when appropriately analyzed and interpreted. While longer-term data is ideal, our findings are consistent with existing research and contribute meaningfully to the field.

*3. This manuscript is recommended for publication in local journals.*

**The author’s answer:**

Thank you for your feedback and for considering our manuscript for publication. We believe that our study offers significant insights into selecting of superior wild hazelnut provenance. The findings have broader implications beyond local contexts and contribute to global tree breeding practices. We believe that the results could benefit a wide audience and stimulate further research in tree breeding. We are open to suggestions on how to further enhance the manuscript to meet the standards of international journals. We are committed to addressing any additional concerns or suggestions to improve the quality and impact of our work. Thank you again for your thoughtful review. We hope that the revisions and additional information provided demonstrate the broader relevance and impact of our research, warranting consideration for publication in this journal.

**Reviewer #3:**

*1. Some sections specially materials and methods as well as the results are too lengthy and needs to be summarized.*

**The author’s answer:**

Thank you very much for bringing that up. We have summarized and modified some parts of the materials and methods and results, hoping to meet your requirements. Due to too many changes in the result part, in order to avoid a long reply, please see the revised manuscript for details (roughly located on pages 5-21, lines 107-335, highlighted in red).

*2. The overall English is good but still needs to be improved preferably by a native speaker.*

**The author’s answer:**

We have done our best to polish the paper, hoping to meet the English level requirements of the journal. If further modifications are needed, we will revise them again in detail.

*3. As mentioned in introduction as well as in table 1, the all plant materials belongs to two distinct species, this is while authors speak about 8 distinct species!! it seems they need to name the plant materials belonged to one species as populations, ecotype etc.*

**The author’s answer:**

Thank you for pointing out the discrepancy. We understand the confusion regarding the classification of the plant materials in our study. In our research, the term "species" was used in a broader sense to describe the different groups of hazelnut plants based on their geographic provenance and distinctive traits. Specifically, we focused on two primary species:

Corylus mandshurica: We collected seeds from this species only from the Nenjiang region.

Corylus heterophylla: Seeds were collected from several regions, including Tieli, Nehe (with two distinct types: one with a striped fruiting testa), Aihui, Longjiang, Mudanjiang, and Xunke.

The term "8 distinct species" was a miscommunication. What we intended to convey is that within the two primary species, we selected materials from different geographic provenances, which are better described as populations rather than separate species. Thus, the eight groups we referred to are actually populations within the two species. Here’s a revised summary to better align with the classification: [The eight populations of the two species come from seven provenances. Corylus mandshurica: One population from Nenjiang. Corylus heterophylla: Seven different populations from Tieli, Nehe (with a variant having a striped fruiting testa), Aihui, Longjiang, Mudanjiang, and Xunke] (page 6, line 116-119).

We have revised the manuscript to reflect this clarification, ensuring that populations is correctly referred to instead of using the term "species" inappropriately. In the revised manuscript, we have changed many "species" into "populations" and marked them in red. Thank you for bringing this to our attention.

*4. Some terms are not selected properly; for example growth condition (instead you can use growth attributes/ or parameters), long in table 2 (instead use length), flowering length in table 3 and 4 (use* *flowering period/ or duration), percent kernel (use* *kernel percentage) etc.*

**The author’s answer:**

Thank you for pointing this out. Based on your comments, we have revised the manuscript. We have changed multiple growth conditions to growth parameters. We have changed long to length in table 2 and change flowering length to flowering period in table 3 and 4. We also have changed multiple percent kernel to kernel percentage. We have marked the revised text in red in Revised Manuscript with Track Changes.

*5. The line for NJ is absent in Fig 2 & 3.*

**The author’s answer:**

Thank you for raising this point. We apologize for not clarifying in the diagram notes why there is no line for NJ. As noted in the explanations for Tables 3 and 4, this is because NJ is not flowering. We have added the explanation "Note: NJ is not flowering" below Figures 1 and 2 in the revised manuscript.

*6. For mean comparison in tables use same order of alphabet letters from highest value to lowest value (a/ or A for highest value).*

**The author’s answer:**

Thank you for your valuable suggestions. We have revised the mean comparison in the table 5, table 6 and table 7, using the same order of the letters of the alphabet from highest to lowest (a/ or A for highest).

Thank you very much for your attention and time. Looking forward to hearing from you.

Your sincerely,

Chunyu Guan

September 6, 2024

College of Life Sciences, Agriculture and Forestry, Qiqihar University
