# Supplementary material for: Characteristics of wild hazelnut populations in Northeast China and selection of superior provenances
Source: PLoS One. 2024 Dec 3;19(12):e0313954. doi: 10.1371/journal.pone.0313954 (PMC11614255; doi:10.1371/journal.pone.0313954)
Supplement: Attachment — Submitted filename: Response to Reviewers.docx [file pone.0313954.s007.docx]

**Dear Editors and Reviewers,**

Thank you very much for your comments and professional advice concerning our manuscript entitled “Characteristics of Wild Hazelnut Populations in Northeast China and Selection of Superior Provenances” (ID: PONE-D-24-23349R2). Your comments are valuable and very helpful for revising and improving the academic rigor of our article, as well as providing significant guidance for our research. We have studied the comments carefully and have made corrections, which we hope will meet with your approval. Furthermore, we would like to present the details as follows:

**Academic Editor：**

*Please review your reference list to ensure that it is complete and correct. If you have cited papers that have been retracted, please include the rationale for doing so in the manuscript text, or remove these references and replace them with relevant current references. Any changes to the reference list should be mentioned in the rebuttal letter that accompanies your revised manuscript. If you need to cite a retracted article, indicate the article’s retracted status in the References list and also include a citation and full reference for the retraction notice.*

**The author’s answer:**

Thank you for your comments. We have revised our references in accordance with the journal's reference format requirements. The following are the details of the revision of the reference (The following numbers are reference numbers, with changes highlighted in red in the revised manuscript):

**[2]:** We added the number of pages. From[Brown JA, Beatty GE, Montgomery WI, Provan J. Broad-scale genetic homogeneity in natural populations of common hazel (Corylus avellana) in Ireland. Tree Genet Genomes. 2016;12(6). doi: ARTN 12210.1007/s11295-016-1079-7. PubMed PMID: WOS:000397238800022.] to [Brown JA, Beatty GE, Montgomery WI, Provan J. Broad-scale genetic homogeneity in natural populations of common hazel (Corylus avellana) in Ireland. Tree Genet Genomes. 2016;12(6):122. doi: 10.1007/s11295-016-1079-7. PubMed PMID: WOS:000397238800022].

**[5]:** We made the changes according to the requirements of the journal reference format. From[Zong JW. Study on Population Genetic Diversity of three Chinese Corylus Species and the Phylogentic Relationship of Corylus Species [PhD]: Chinese Academy of Forestry; 2017] to [Zong JW. Study on Population Genetic Diversity of three Chinese Corylus Species and the Phylogentic Relationship of Corylus Species. PhD. Thesis, Chinese Academy of Forestry; 2017. Available from: <https://kns.cnki.net/kcms2/article/abstract?v=_W1AupcyYgZJYRJtAWj7Rty7HrHEdpbxgn9Qg1m4Msdu9fvGGxf9aUbhf2a3hQcSgkSxk4JKLgzHWheDOdXcswqGUsQHPO-ao1virqxCWge0oY-54IJw4MYaJ3DkWGTFAmDqHWWm1FhHUVd6nhN8dLBylgj1YZ4t5VzU51_e_NBlxQAHapTAcZPaD_WUuDDDfWXnsjfLcyI=&uniplatform=NZKPT&language=CHS>].

**[12]:** We added DOI. From[Luo F, Fei XQ, Tang FB, Li XL. Simultaneous determination of paclitaxel in hazelnut by HPLC-MS/MS. Forest Research. 2011;24:779-83] to [Luo F, Fei XQ, Tang FB, Li XL. Simultaneous determination of paclitaxel in hazelnut by HPLC-MS/MS. Forest Research. 2011;24:779-83. doi: <https://doi.org/10.1016/j.heliyon.2023.e13675>].

**[17]:** We made the changes in accordance with the format requirements of the reference "Book chapters" in the journal. From[Vahdati K, Sheikhi A, Arab MM, Sarikhani S, Habibi A, Ataee H. Cultivars and Genetic Improvement. In: Mir MM, Rehman MU, Iqbal U, Mir SA, editors. Temperate Nuts. Singapore: Springer Nature Singapore; 2023. p. 79-111] to [Vahdati K, Sheikhi A, Arab MM, Sarikhani S, Habibi A, Ataee H. Cultivars and Genetic Improvement. In: Mir MM, Rehman MU, Iqbal U, Mir SA, editors. Temperate Nuts. Singapore: Springer Nature Singapore; 2023. pp. 79-111].

**[18]:** We have added journal names, volumes, pages and DOI. From[Vahdati K, Khorami SS, editors. The past, present and future of walnut genetic improvement and propagation2021: International Society for Horticultural Science (ISHS), Leuven, Belgium] to [Vahdati K, Khorami SS, editors. The past, present and future of walnut genetic improvement and propagation. Acta Hortic. 1318: 251-258. doi: <https://doi.org/10.17660/ActaHortic.2021.1318.37>].

**[19]:** We made the changes in accordance with the format requirements of the reference "Book chapters" in the journal. From[Vahdati K, Arab M, Sarikhani S, Sadat-Hosseini M, Leslie C, Brown P. Advances in Persian Walnut (Juglans regia L.) Breeding Strategies. 2019. p. 401-72] to [Vahdati K, Arab M, Sarikhani S, Sadat-Hosseini M, Leslie C, Brown P. In: Advances in Persian Walnut (Juglans regia L.) Breeding Strategies. Al-Khayri, J., Jain, S., Johnson, D. (eds) Advances in Plant Breeding Strategies: Nut and Beverage Crops. Springer, Cham; 2019. pp. 401-72].

**[25]:** We added pages. From[Zhao T, Ma W, Yang Z, Liang L, Chen X, Wang G, et al. A chromosome-level reference genome of the hazelnut, Corylus heterophylla Fisch. Gigascience. 2021;10(4). Epub 2021/04/20. doi: 10.1093/gigascience/giab027. PubMed PMID: 33871007; PubMed Central PMCID: PMCPMC8054262] to [Zhao T, Ma W, Yang Z, Liang L, Chen X, Wang G, et al. A chromosome-level reference genome of the hazelnut, Corylus heterophylla Fisch. Gigascience. 2021;10(4): 1-9 Epub 2021/04/20. doi: 10.1093/gigascience/giab027. PubMed PMID: 33871007; PubMed Central PMCID: PMCPMC8054262].

**[29]:** We have added journal names, volumes, pages and DOI. From[Vahdati K, Mohseniazar M, editors. Early bearing genotypes of walnut: a suitable material for breeding and high density orchards2016: International Society for Horticultural Science (ISHS), Leuven, Belgium] to [Vahdati K, Mohseniazar M, editors. Early bearing genotypes of walnut: a suitable material for breeding and high density orchards. Acta Hortic. 1139: 101-106. doi: <https://doi.org/10.17660/ActaHortic.2016.1139.18>].

**[30]:** We have added journal names, volumes, pages and DOI. From[Vahdati K, Karimi R, Ershadi A, editors. GENETIC STRUCTURE OF SOME WILD WALNUT POPULATIONS IN IRAN2015: International Society for Horticultural Science (ISHS), Leuven, Belgium] to [Vahdati K, Karimi R, Ershadi A, editors. GENETIC STRUCTURE OF SOME WILD WALNUT POPULATIONS IN IRAN. Acta Hortic. 1074: 125-128. doi: <https://doi.org/10.17660/ActaHortic.2015.1074.18>]

**[34]:** We are sorry that we did not find the DOI of this reference. But we have provided the link here: <https://kns.cnki.net/kcms2/article/abstract?v=_W1AupcyYgZACCJ7g5T4YcpuIJNx0YaDr-dpt2j_vUqw28MX81RkRe2ARt84fqqCapqeVWZKnPHbRwU22_l7vVtPkwdAXJy2TSWpUQxHh-JjQEWzHfpG2GaU-Ul5OAJ0suup9_2ie4FJ5iMF8yqTsKKCPnF8gff7uNQlT7gemRg0L4_nuRm1Qhnwt8v8FP_P&uniplatform=NZKPT&language=CHS>.

**[35]:** We added DOI. From[Long ZY, Lu CH. Resourse distribution and development research progress of Corylus in Heilongjiang province. Forest By-Product and Speciality in China. 2005;(04):41-2] to [Long ZY, Lu CH. Resourse distribution and development research progress of Corylus in Heilongjiang province. Forest By-Product and Speciality in China. 2005;(04):41-42. doi: :10.13268/j.cnki.fbsic.2005.04.030].

**[45]:** We revised the references according to the requirements in the journal reference format "Masters' theses or doctoral dissertations". From[WANG WB. Florescence Phenology and Techniques for Promoting Blossom and Fruiting of Corylus spp. [Master]: Northeast Forestry University; 2002] to [WANG WB. Florescence Phenology and Techniques for Promoting Blossom and Fruiting of Corylus spp. M.Sc. Thesis, Northeast Forestry University; 2002. Available from: <https://kns.cnki.net/kcms2/article/abstract?v=_W1AupcyYgY_l3KMaGPX-H9LOGYFEl9x2B7urTOoRP_AnWmCXaTeE62_AN5hQW80-UczQml1K-YQTt-PJ0v4o1xUwijY9mSxG8QejPDA4nqiGwt3NINHEOKLAzTfFX6E4LUrsMZ5wzQL6rWwKetwN-4GhsztS4oy0RfhaDb_vK9aADjXuTFKNIv-nNsuf9Pn&uniplatform=NZKPT&language=CHS>].

**[46]:** We revised the references according to the requirements in the journal reference format "Masters' theses or doctoral dissertations". From[Wang Q. Study on the biological characteristics of flowering and fruiting of hazelnut hybrid [Master]: Xinjiang Agricultural University; 2022] to [Wang Q. Study on the biological characteristics of flowering and fruiting of hazelnut hybrid. M.Sc. Thesis, Xinjiang Agricultural University; 2022. Available from: <https://kns.cnki.net/kcms2/article/abstract?v=_W1AupcyYgY5BVeZhTs5dAw8WvT_Qn9jb8BkPiDd7qmLnqxr8oCR83NLX3-DuCs6EOWOFdmc5kGw0CDYdnfX8rodohgUbyb_P0MeQwSFE9_pchtjk0tatb7cdDCwjUMrbExGoSPSRZiDHrllB6d6pW32qiZ2wBzjcpAlPteWKohGX7iAnbQPJ9tCp4Rj_9buflbuX_WVjjg=&uniplatform=NZKPT&language=CHS>].

**[47]:** We are sorry that we did not find the DOI of this reference. But we have provided the link here: <https://kns.cnki.net/kcms2/article/abstract?v=_W1AupcyYgb813IaIi9LDlH_JKnWS4CNXgPCigdAjfQaBHFPMQweE4viS_iMn8P29SyXRAGdPzRzeniRyJ5UCt_-A5vxyE-puwj32dbPYZ3vZTBgpq1VROorCmb6kqCVYGJo7VY6-k1Fj6hVXzUVu-Sc7RgXkmLJeYd0n3MfyYZMk0zQgd0n4eyU6sgBlWAg&uniplatform=NZKPT&language=CHS>.

**[48]:** We are sorry that we did not find the DOI of this reference. But we have provided the link here: <https://kns.cnki.net/kcms2/article/abstract?v=_W1AupcyYgbBBbNdvDZM7u4fc2TSHUn37i6Sjk7ds3RkcPAyXpYfvCNhWUKrmY-Ode0-UVHDpSiF2Zf_GqjUehT4nJUuOkUmQYJCBe8R0-WDwgoFQRnifw7d_eW2L5vnXW71aT5F2sR2AEwkX-1qX0C39MzQBmXRNLe8hhaB7XA5kDFMxkkCYKAQgdl-e7AL&uniplatform=NZKPT&language=CHS>.

**[49]:** We added DOI. From[Nikolaieva N, Brindza J, Garkava K, Ostrovský R. Pollen features of hazelnut (Corylus avellana L.) from different habitats. Modern Phytomorphology. 2014;6:53-8] to [Nikolaieva N, Brindza J, Garkava K, Ostrovský R. Pollen features of hazelnut (Corylus avellana L.) from different habitats. Modern Phytomorphology. 2014;6:53-8. doi: :10.5281/zenodo.160440].

**[50]:** We added DOI. From[Li JJ, Zhang R, Q., Ma QH, Wang GX. SEM observation on the pollen morphology in Corylus. Journal of Chinese Electron Microscopy Society. 2017;36(04):404-13] to [Li JJ, Zhang R, Q., Ma QH, Wang GX. SEM observation on the pollen morphology in Corylus. Journal of Chinese Electron Microscopy Society. 2017;36(04):404-13. doi: 10.3969/j.issn.1000-6281.2017.04.014].

**[56]:** We added DOI. From[Matthäus B, Özcan MM. The comparison of properties of the oil and kernels of various hazelnuts from Germany and Turkey. European Journal of Lipid Science and Technology. 2012;114:801-6] to [Matthäus B, Özcan MM. The comparison of properties of the oil and kernels of various hazelnuts from Germany and Turkey. European Journal of Lipid Science and Technology. 2012;114:801-6. doi: <https://doi.org/10.1002/ejlt.201100299>].

**Reviewer #1:**

1. *Reference Formatting: As you pointed out, some of the newly added references do not include the journal names, page numbers, or volumes. The authors should standardize their references in line with the required citation style of PLOS ONE. Ensuring complete and consistent referencing, including missing elements (e.g., journal name, pages, volume), is critical for a high-quality academic manuscript.*

**The author’s answer:**

The following are the details of the revision of the reference (The following numbers are reference numbers, with changes highlighted in red in the revised manuscript):

**[2]:** We added the number of pages. From[Brown JA, Beatty GE, Montgomery WI, Provan J. Broad-scale genetic homogeneity in natural populations of common hazel (Corylus avellana) in Ireland. Tree Genet Genomes. 2016;12(6). doi: ARTN 12210.1007/s11295-016-1079-7. PubMed PMID: WOS:000397238800022.] to [Brown JA, Beatty GE, Montgomery WI, Provan J. Broad-scale genetic homogeneity in natural populations of common hazel (Corylus avellana) in Ireland. Tree Genet Genomes. 2016;12(6):122. doi: 10.1007/s11295-016-1079-7. PubMed PMID: WOS:000397238800022].

**[5]:** We made the changes according to the requirements of the journal reference format. From[Zong JW. Study on Population Genetic Diversity of three Chinese Corylus Species and the Phylogentic Relationship of Corylus Species [PhD]: Chinese Academy of Forestry; 2017] to [Zong JW. Study on Population Genetic Diversity of three Chinese Corylus Species and the Phylogentic Relationship of Corylus Species. PhD. Thesis, Chinese Academy of Forestry; 2017. Available from: <https://kns.cnki.net/kcms2/article/abstract?v=_W1AupcyYgZJYRJtAWj7Rty7HrHEdpbxgn9Qg1m4Msdu9fvGGxf9aUbhf2a3hQcSgkSxk4JKLgzHWheDOdXcswqGUsQHPO-ao1virqxCWge0oY-54IJw4MYaJ3DkWGTFAmDqHWWm1FhHUVd6nhN8dLBylgj1YZ4t5VzU51_e_NBlxQAHapTAcZPaD_WUuDDDfWXnsjfLcyI=&uniplatform=NZKPT&language=CHS>].

**[12]:** We added DOI. From[Luo F, Fei XQ, Tang FB, Li XL. Simultaneous determination of paclitaxel in hazelnut by HPLC-MS/MS. Forest Research. 2011;24:779-83] to [Luo F, Fei XQ, Tang FB, Li XL. Simultaneous determination of paclitaxel in hazelnut by HPLC-MS/MS. Forest Research. 2011;24:779-83. doi: <https://doi.org/10.1016/j.heliyon.2023.e13675>].

**[17]:** We made the changes in accordance with the format requirements of the reference "Book chapters" in the journal. From[Vahdati K, Sheikhi A, Arab MM, Sarikhani S, Habibi A, Ataee H. Cultivars and Genetic Improvement. In: Mir MM, Rehman MU, Iqbal U, Mir SA, editors. Temperate Nuts. Singapore: Springer Nature Singapore; 2023. p. 79-111] to [Vahdati K, Sheikhi A, Arab MM, Sarikhani S, Habibi A, Ataee H. Cultivars and Genetic Improvement. In: Mir MM, Rehman MU, Iqbal U, Mir SA, editors. Temperate Nuts. Singapore: Springer Nature Singapore; 2023. pp. 79-111].

**[18]:** We have added journal names, volumes, pages and DOI. From[Vahdati K, Khorami SS, editors. The past, present and future of walnut genetic improvement and propagation2021: International Society for Horticultural Science (ISHS), Leuven, Belgium] to [Vahdati K, Khorami SS, editors. The past, present and future of walnut genetic improvement and propagation. Acta Hortic. 1318: 251-258. doi: <https://doi.org/10.17660/ActaHortic.2021.1318.37>].

**[19]:** We made the changes in accordance with the format requirements of the reference "Book chapters" in the journal. From[Vahdati K, Arab M, Sarikhani S, Sadat-Hosseini M, Leslie C, Brown P. Advances in Persian Walnut (Juglans regia L.) Breeding Strategies. 2019. p. 401-72] to [Vahdati K, Arab M, Sarikhani S, Sadat-Hosseini M, Leslie C, Brown P. In: Advances in Persian Walnut (Juglans regia L.) Breeding Strategies. Al-Khayri, J., Jain, S., Johnson, D. (eds) Advances in Plant Breeding Strategies: Nut and Beverage Crops. Springer, Cham; 2019. pp. 401-72].

**[25]:** We added pages. From[Zhao T, Ma W, Yang Z, Liang L, Chen X, Wang G, et al. A chromosome-level reference genome of the hazelnut, Corylus heterophylla Fisch. Gigascience. 2021;10(4). Epub 2021/04/20. doi: 10.1093/gigascience/giab027. PubMed PMID: 33871007; PubMed Central PMCID: PMCPMC8054262] to [Zhao T, Ma W, Yang Z, Liang L, Chen X, Wang G, et al. A chromosome-level reference genome of the hazelnut, Corylus heterophylla Fisch. Gigascience. 2021;10(4): 1-9 Epub 2021/04/20. doi: 10.1093/gigascience/giab027. PubMed PMID: 33871007; PubMed Central PMCID: PMCPMC8054262].

**[29]:** We have added journal names, volumes, pages and DOI. From[Vahdati K, Mohseniazar M, editors. Early bearing genotypes of walnut: a suitable material for breeding and high density orchards2016: International Society for Horticultural Science (ISHS), Leuven, Belgium] to [Vahdati K, Mohseniazar M, editors. Early bearing genotypes of walnut: a suitable material for breeding and high density orchards. Acta Hortic. 1139: 101-106. doi: <https://doi.org/10.17660/ActaHortic.2016.1139.18>].

**[30]:** We have added journal names, volumes, pages and DOI. From[Vahdati K, Karimi R, Ershadi A, editors. GENETIC STRUCTURE OF SOME WILD WALNUT POPULATIONS IN IRAN2015: International Society for Horticultural Science (ISHS), Leuven, Belgium] to [Vahdati K, Karimi R, Ershadi A, editors. GENETIC STRUCTURE OF SOME WILD WALNUT POPULATIONS IN IRAN. Acta Hortic. 1074: 125-128. doi: <https://doi.org/10.17660/ActaHortic.2015.1074.18>]

**[34]:** We are sorry that we did not find the DOI of this reference. But we have provided the link here: <https://kns.cnki.net/kcms2/article/abstract?v=_W1AupcyYgZACCJ7g5T4YcpuIJNx0YaDr-dpt2j_vUqw28MX81RkRe2ARt84fqqCapqeVWZKnPHbRwU22_l7vVtPkwdAXJy2TSWpUQxHh-JjQEWzHfpG2GaU-Ul5OAJ0suup9_2ie4FJ5iMF8yqTsKKCPnF8gff7uNQlT7gemRg0L4_nuRm1Qhnwt8v8FP_P&uniplatform=NZKPT&language=CHS>.

**[35]:** We added DOI. From[Long ZY, Lu CH. Resourse distribution and development research progress of Corylus in Heilongjiang province. Forest By-Product and Speciality in China. 2005;(04):41-2] to [Long ZY, Lu CH. Resourse distribution and development research progress of Corylus in Heilongjiang province. Forest By-Product and Speciality in China. 2005;(04):41-42. doi: :10.13268/j.cnki.fbsic.2005.04.030].

**[45]:** We revised the references according to the requirements in the journal reference format "Masters' theses or doctoral dissertations". From[WANG WB. Florescence Phenology and Techniques for Promoting Blossom and Fruiting of Corylus spp. [Master]: Northeast Forestry University; 2002] to [WANG WB. Florescence Phenology and Techniques for Promoting Blossom and Fruiting of Corylus spp. M.Sc. Thesis, Northeast Forestry University; 2002. Available from: <https://kns.cnki.net/kcms2/article/abstract?v=_W1AupcyYgY_l3KMaGPX-H9LOGYFEl9x2B7urTOoRP_AnWmCXaTeE62_AN5hQW80-UczQml1K-YQTt-PJ0v4o1xUwijY9mSxG8QejPDA4nqiGwt3NINHEOKLAzTfFX6E4LUrsMZ5wzQL6rWwKetwN-4GhsztS4oy0RfhaDb_vK9aADjXuTFKNIv-nNsuf9Pn&uniplatform=NZKPT&language=CHS>].

**[46]:** We revised the references according to the requirements in the journal reference format "Masters' theses or doctoral dissertations". From[Wang Q. Study on the biological characteristics of flowering and fruiting of hazelnut hybrid [Master]: Xinjiang Agricultural University; 2022] to [Wang Q. Study on the biological characteristics of flowering and fruiting of hazelnut hybrid. M.Sc. Thesis, Xinjiang Agricultural University; 2022. Available from: <https://kns.cnki.net/kcms2/article/abstract?v=_W1AupcyYgY5BVeZhTs5dAw8WvT_Qn9jb8BkPiDd7qmLnqxr8oCR83NLX3-DuCs6EOWOFdmc5kGw0CDYdnfX8rodohgUbyb_P0MeQwSFE9_pchtjk0tatb7cdDCwjUMrbExGoSPSRZiDHrllB6d6pW32qiZ2wBzjcpAlPteWKohGX7iAnbQPJ9tCp4Rj_9buflbuX_WVjjg=&uniplatform=NZKPT&language=CHS>].

**[47]:** We are sorry that we did not find the DOI of this reference. But we have provided the link here: <https://kns.cnki.net/kcms2/article/abstract?v=_W1AupcyYgb813IaIi9LDlH_JKnWS4CNXgPCigdAjfQaBHFPMQweE4viS_iMn8P29SyXRAGdPzRzeniRyJ5UCt_-A5vxyE-puwj32dbPYZ3vZTBgpq1VROorCmb6kqCVYGJo7VY6-k1Fj6hVXzUVu-Sc7RgXkmLJeYd0n3MfyYZMk0zQgd0n4eyU6sgBlWAg&uniplatform=NZKPT&language=CHS>.

**[48]:** We are sorry that we did not find the DOI of this reference. But we have provided the link here: <https://kns.cnki.net/kcms2/article/abstract?v=_W1AupcyYgbBBbNdvDZM7u4fc2TSHUn37i6Sjk7ds3RkcPAyXpYfvCNhWUKrmY-Ode0-UVHDpSiF2Zf_GqjUehT4nJUuOkUmQYJCBe8R0-WDwgoFQRnifw7d_eW2L5vnXW71aT5F2sR2AEwkX-1qX0C39MzQBmXRNLe8hhaB7XA5kDFMxkkCYKAQgdl-e7AL&uniplatform=NZKPT&language=CHS>.

**[49]:** We added DOI. From[Nikolaieva N, Brindza J, Garkava K, Ostrovský R. Pollen features of hazelnut (Corylus avellana L.) from different habitats. Modern Phytomorphology. 2014;6:53-8] to [Nikolaieva N, Brindza J, Garkava K, Ostrovský R. Pollen features of hazelnut (Corylus avellana L.) from different habitats. Modern Phytomorphology. 2014;6:53-8. doi: :10.5281/zenodo.160440].

**[50]:** We added DOI. From[Li JJ, Zhang R, Q., Ma QH, Wang GX. SEM observation on the pollen morphology in Corylus. Journal of Chinese Electron Microscopy Society. 2017;36(04):404-13] to [Li JJ, Zhang R, Q., Ma QH, Wang GX. SEM observation on the pollen morphology in Corylus. Journal of Chinese Electron Microscopy Society. 2017;36(04):404-13. doi: 10.3969/j.issn.1000-6281.2017.04.014].

**[56]:** We added DOI. From[Matthäus B, Özcan MM. The comparison of properties of the oil and kernels of various hazelnuts from Germany and Turkey. European Journal of Lipid Science and Technology. 2012;114:801-6] to [Matthäus B, Özcan MM. The comparison of properties of the oil and kernels of various hazelnuts from Germany and Turkey. European Journal of Lipid Science and Technology. 2012;114:801-6. doi: <https://doi.org/10.1002/ejlt.201100299>].

2. ***Clarification on One-Year Data: Reviewer #2 questioned the reliability of one-year measurement data, advising a longer observational period. The authors justified their use of one-year data by referencing another publication, but they should be clearer about potential limitations and perhaps indicate plans for longer-term data collection in future studies to strengthen this defense.***

**The author’s answer:**

In response to this question, we indicate potential limitations of the study in the "discussion" section of the article and indicate plans for long-term data collection in future studies [This study observed only one year of growth due to time constraints. However, in some research, a one-year growth index holds significant reference value [53]. Future studies should aim for long-term data collection to enhance the stability of growth index analysis results] (page 24, line 400-403).

3. ***Potential for Further Summarization: While the authors shortened some sections, there are still lengthy parts, particularly in the results section. More concise presentation could enhance the paper’s readability.***

**The author’s answer:**

1. We have reduced the repetition of what is already mentioned in the table and removed redundant statements that do not summarize the results effectively, including the following changes:
(1) From [Analysis revealed that NH1 had the longest male inflorescence length both before and after opening, while TL exhibited the shortest. Before opening, TL had the widest male inflorescence at 3.5 mm, whereas NH2 had the narrowest at 3.2 mm. After opening, LJ had the widest inflorescence at 3.6 mm, and AH had the narrowest at 2.8 mm] to [Analysis revealed that NH1 had the longest male inflorescence length both before and after opening. Before opening, TL had the widest male inflorescence at 3.5 mm, while after opening, LJ had the widest inflorescence at 3.6 mm.] (page 10, line 198-200).
(2) From [The initial flowering stage for all populations began on April 2, with no significant differences. NH1, NH2, and MDJ reached the blooming stage by April 7, whereas AH began blooming on April 10. NH1 and NH2 were the earliest to reach the end stage on April 13, with the shortest flowering period of 12 days, while LJ was the latest, reaching the end stage on April 19 and exhibiting the longest flowering period of 18 days] to [The initial flowering stage for all populations began on April 2, with no significant differences noted. NH1 and NH2 exhibited the shortest flowering period of 12 days, whereas LJ displayed the longest period of 18 days] (page 11, line 209-212).
(3) From [Comparison of female flowering stage observations of eight hazelnut populations was shown in Table 4. From the tables, it could be seen that NH2 and MDJ entered the initial flowering stage (April 2) and blooming stage (April 9) the earliest, and XK entered the initial flowering stage (April 11) and blooming stage (April 16) the latest. The earliest to enter end stage was MDJ on April 13, with TL, AH, LJ, and XK entering end stage as late as April 22nd. MDJ and XK had the shortest florescence of 12d and NH2 had the longest florescence of 19d] to [Table 4 presents a comparison of female flowering stage observations among eight hazelnut populations. MDJ and XK exhibited the shortest florescence of 12 days, while NH2 had the longest florescence at 19 days] (page 12, line 224-226).
(4) From [Each grain had three adjacent germination apertures, with varying degrees of invagination depending on the populations. Table 5 presents the pollen measurements, showing polar axis lengths ranging from 14.02 to 17.09 μm, equatorial axis lengths from 16.02 to 18.75 μm, and a polar axis/equatorial axis (P/E) ratio from 0.88 to 0.92 μm] to [Each grain possessed three adjacent germination apertures. Table 5 presents the pollen measurements, indicating polar axis lengths ranging from 14.02 to 17.09 μm and equatorial axis lengths from 16.02 to 18.75 μm] (page 13, line 241-243).
(5) From [The analysis showed that after the year's growth, MDJ had the highest plant height of 112.53 cm and NH2 had the largest ground diameter of 13.74 cm, while XK had a plant height of 90.20 cm and a ground diameter of 9.16 mm, which was the smallest among these six hazelnut populations. There was a highly significant difference in plant height (P < 0.01)] to [The analysis showed that after the year's growth, MDJ had the highest plant height of 112.53 cm and NH2 had the largest ground diameter of 13.74 cm. There was a highly significant difference in plant height (P < 0.01)] (page 14-15, line 255-257).
(6) From [Taking the eigenvalue of principal components greater than 1 as the standard, a total of 2 principal components were extracted: the eigenvalue of principal component 1 was 4.136, and the variance contribution rate was 55.127%, which could represent 55.127% of the total trait information, and the factors determining the size of the 1st principal component were mainly the nut thickness and the nut length, which were loaded with larger loadings of 0.895 and 0.881, respectively, reflecting the size of the nut. The eigenvalue of principal component 2 was 1.687, and the variance contribution rate was 22.489%, and the factors determining the size of the 2nd principal component were kernel percentage and kernel weight, whose loadings were 1.016 and 0.458, respectively, reflecting the nut yield] to [Using an eigenvalue threshold greater than 1 as the standard, a total of two principal components were extracted. The variance contribution rate for the first principal component was 55.127%, representing 55.127% of the total trait information. The primary factors influencing the first principal component were nut thickness and nut length, which reflect the size of the nut. The variance contribution rate for the second principal component was 22.489%, with kernel percentage and kernel weight as the main factors, reflecting nut yield] (page 17, line 295-300).
2. We have also added some concluding sentences, including the following changes:
(1) Differences were observed in the length, width, flowering rate, and openness of male flower sequences among hazelnut varieties. Notably, varieties TL and LJ exhibited prominence across multiple indices (page 10-11, line 203-205).
(2) Overall, the nutritional content of the NH2 kernel is relatively high (page 19, line 330-331).

Thank you very much for your attention and time. We hope our modification can meet your requirements. Looking forward to hearing from you.

Your sincerely,

Chunyu Guan

October 24, 2024

College of Life Sciences, Agriculture and Forestry, Qiqihar University
